# Supplementary figures and images for: Guanylate-binding protein 1 acts as a pro-viral factor for the life cycle of hepatitis C virus
Source: PLoS Pathog. 2024 Feb 5;20(2):e1011976. doi: 10.1371/journal.ppat.1011976 (PMC10868826; doi:10.1371/journal.ppat.1011976)

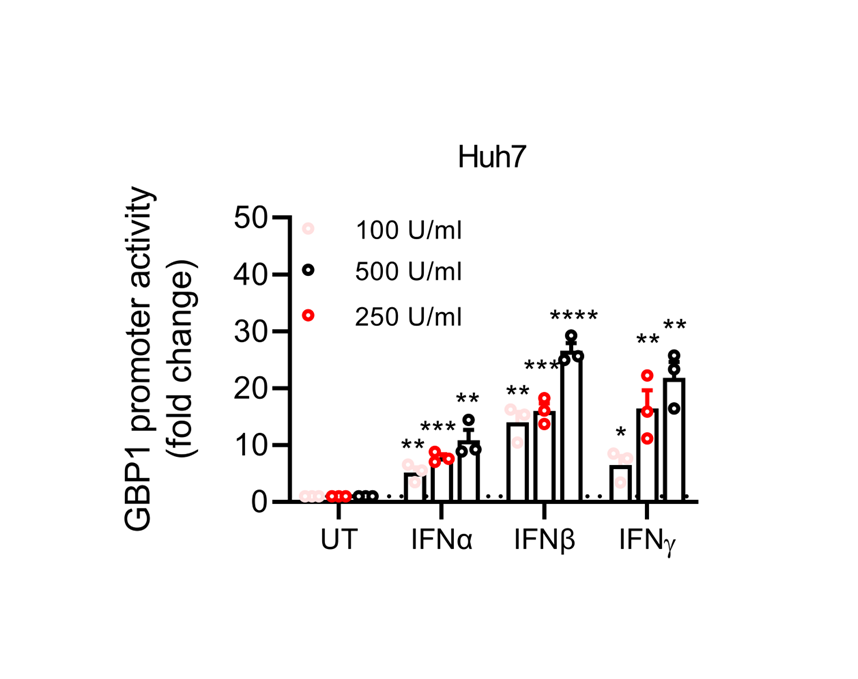

Supplement: S1 Fig — Luciferase reporter gene assay of Huh7 cells transfected with pGL3-GBP1-Luc plasmid DNA (GBP1-Luc) to monitor GBP1 expression after treatment with different concentrations (100 U/ml, 250 U/ml, 500 U/ml) of IFNα, IFNβ, IFNγ for 24 h (48 h post seeding). Relative values are referred to the untreated control (UT) (set to 1). N = 9 technical replicates. (TIF) [file ppat.1011976.s001.tif]

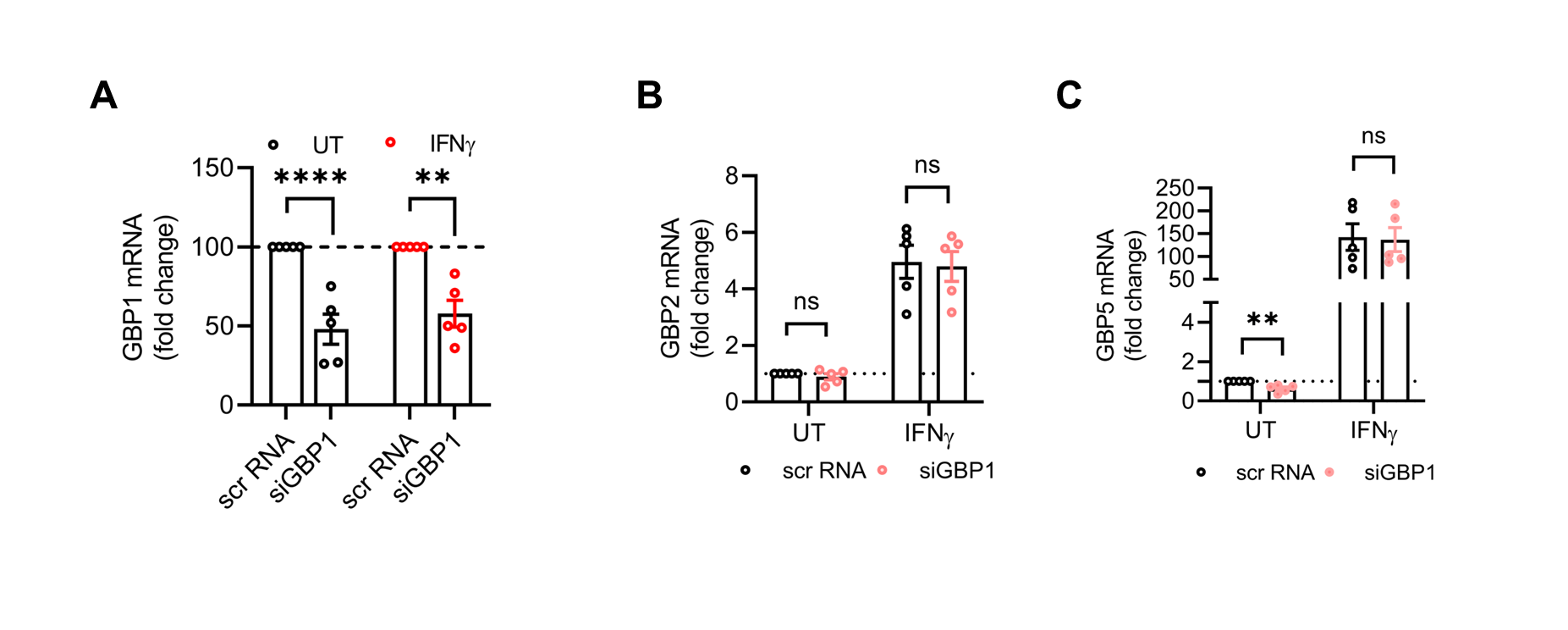

Supplement: S2 Fig — (A) qPCR analysis to monitor GBP1 mRNA levels 72 h after GBP1 silencing in stable HCV positive Huh7.5 cells (Jc1). Graph shows average percent change of the GBP1 levels referred to the untreated (UT) scrambled (scr RNA) control (set to 100). N = 5 biological replicates. Statistics were performed as mean ± SEM, unpaired t-test referred to ctrl. with **p<0.01, ****p<0.0001. (B, C) qPCR analysis to monitor GBP2 (B) and GBP5 (C) mRNA levels 72 h after GBP1 silencing in stable HCV positive cells (Jc1). Relative change of GBP2/5 levels were referred to the untreated (UT) scrambled (scr RNA) control (set to 1). N = 5 biological replicates. Statistics were performed as mean ± SEM, unpaired t-test referred to ctrl. with **p<0.01, ****p<0.0001. (TIF) [file ppat.1011976.s002.tif]

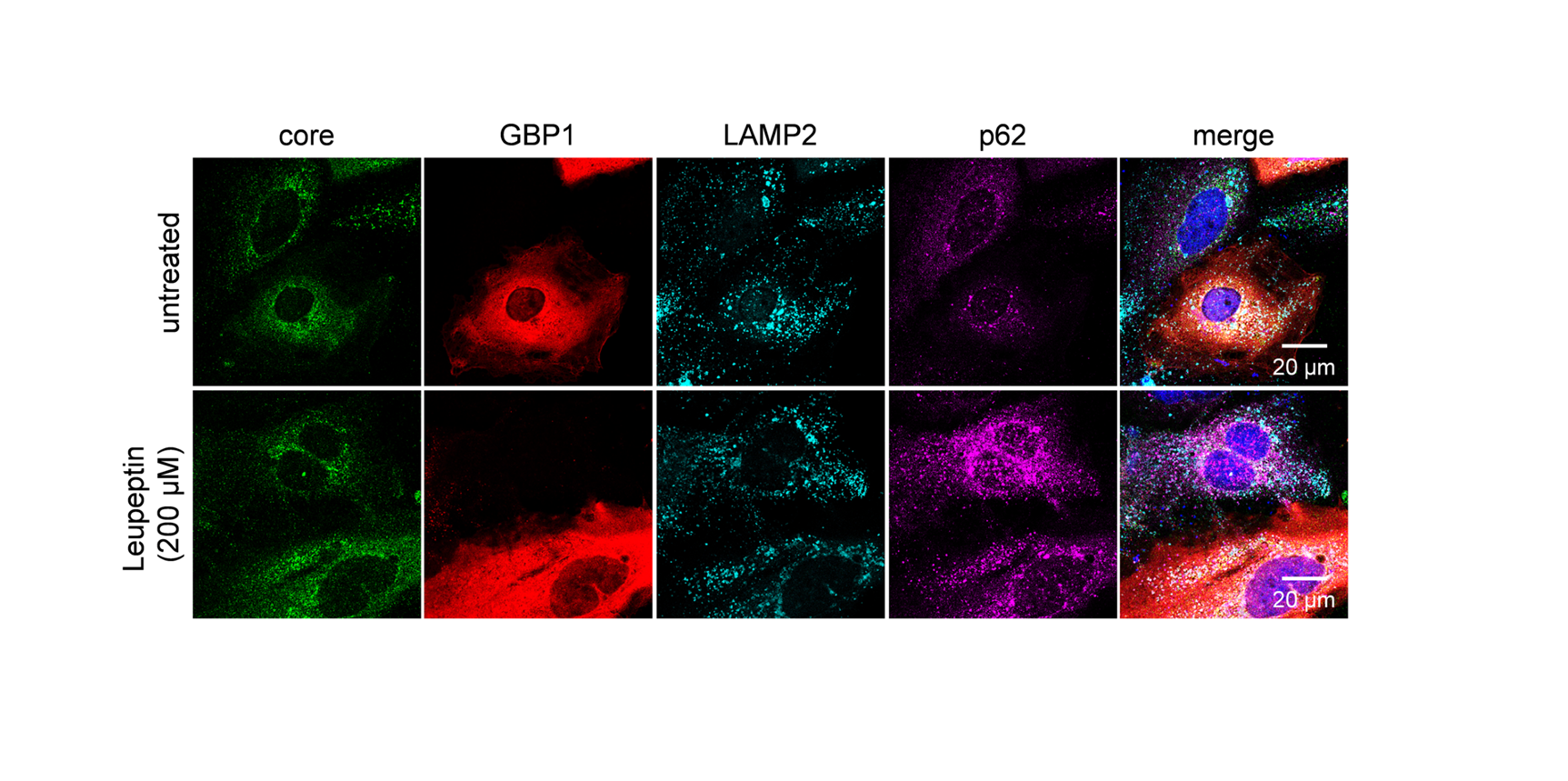

Supplement: S3 Fig — CLSM analysis of stably HCV replicating Huh7.5 cells transfected with a GBP1-Wt encoding construct using GBP1- (red), HCV core (green)-, LAMP2 (cyan) and p62 (magenta)-specific antisera. To inhibit lysosomal degradation the cells were treated with Leupeptin (200μM) for 24 h or left untreated as a control (UT). The cells were analyzed 48 h pt. Scale bars, 20 μm. (TIF) [file ppat.1011976.s003.tif]

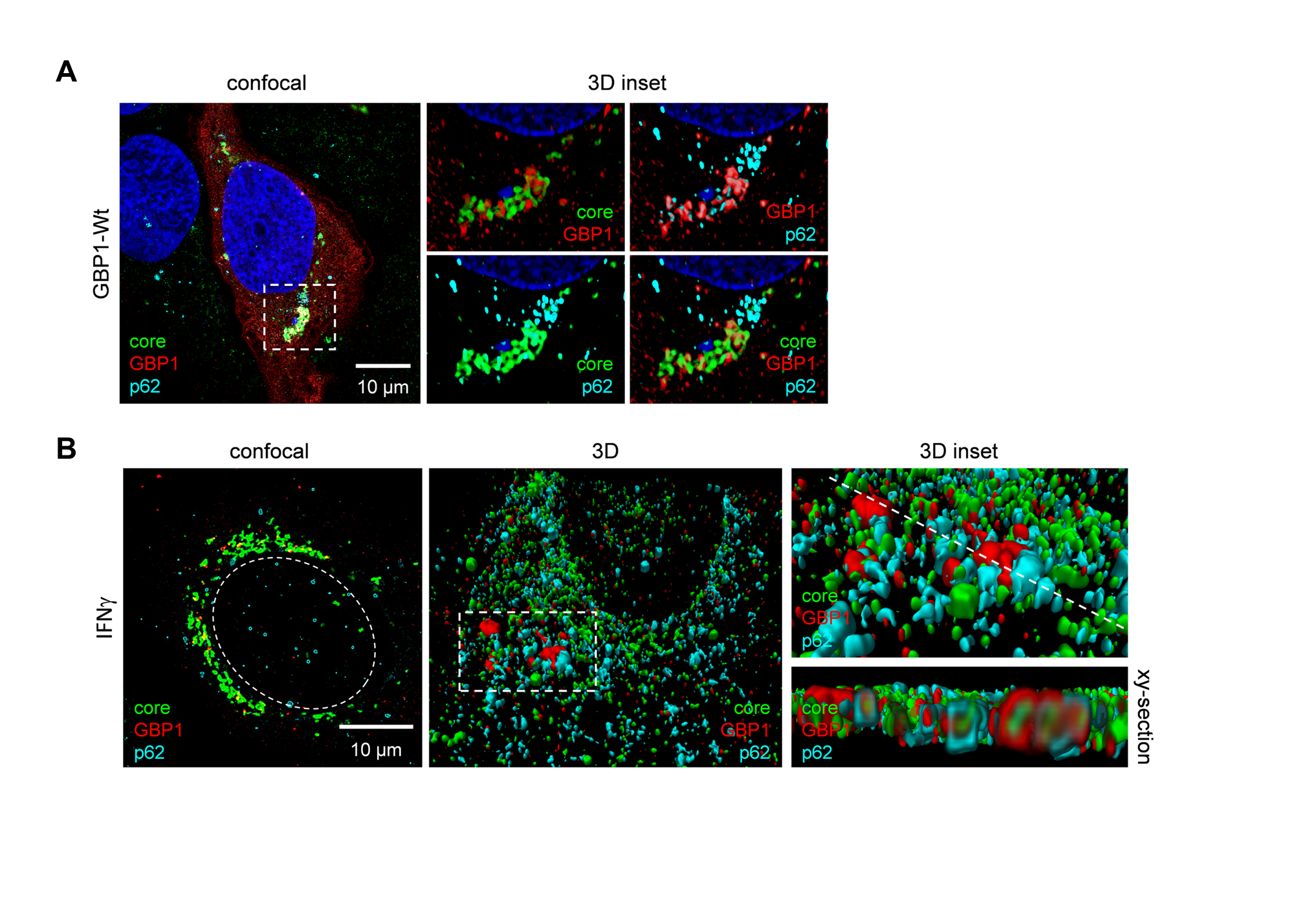

Supplement: S4 Fig — (A) CLSM analysis and 3D reconstructions (3D inset) of stable HCV positive cells (Jc1) to visualize p62 colocalizing with GBP1 and HCV core. 24 h post seeding the cells were transfected with a GBP1-Wt construct for 48 h and fixed with a 1:1 mixture of ice-cold ethanol/acetone for 10 min. The cells were stained for GBP1 (red), core (green), and p62 (cyan) using specific antisera. Nuclei were visualized with DAPI. Scale bar, 10 μm. Images are representative of 3 biological replicates. (B) CLSM analysis, z-stacks and 3D reconstructions (3D inset, 3D xy-sections) of stable HCV positive Huh7.5 cells (Jc1) to visualize p62 colocalizing with GBP1 and HCV core. To induce GBP1 expression the cells were treated with IFNγ (100 U/ml) 24 h post seeding. After 24 h the cells were fixed with a 1:1 mixture of ice-cold ethanol/acetone for 10 min. The cells were stained for GBP1 (red), core (green), and p62 (cyan) using specific antisera. Nuclei were visualized with DAPI. Scale bar, 10 μm. Images are representative of 3 biological replicates. All immunofluorescence images were deconvoluted using the LasX Lightning Tool using the adaptive algorithm. (TIF) [file ppat.1011976.s004.tif]
