# Supplementary material for: Guanylate-binding protein 1 acts as a pro-viral factor for the life cycle of hepatitis C virus
Source: PLoS Pathog. 2024 Feb 5;20(2):e1011976. doi: 10.1371/journal.ppat.1011976 (PMC10868826; doi:10.1371/journal.ppat.1011976)
Supplement: Attachment — Submitted filename: xfi point by point.docx [file ppat.1011976.s005.docx]

**Point-by-point reply PPATHOGENS-D-23-01540 - [EMID:3a094e76119a12cc**]

**Title**

Guanylate-Binding Protein 1 acts as a pro-viral factor for the life cycle of Hepatitis C virus

**Authors**

Daniela Bender ^1^, Alexandra Koulouri ^1^, Xingjian Wen ^3^, Mirco Glitscher ^1^, Anja Schollmeier ^1^, Liliana Fernandes da Costa ^1^, Robin Oliver Murra ^1^, Gert Carra ^1^, Vanessa Haberger ^1^, Gerrit J.K. Praefcke ^2^, and Eberhard Hildt ^1,^*

^1^	Paul-Ehrlich-Institut, Department of Virology, Langen, Germany; ^2^ Paul-Ehrlich-Institut, Department Haematology and Transfusion Medicine Langen, Germany.^3^Chongqing Academy of Chinese Materia Medica, Chongqing 400065, PR China.

* Corresponding author.

**Correspondence**

Prof. Dr. Eberhard Hildt; Paul-Ehrlich-Strasse 51-59, D-63225 Langen, Germany; [eberhard.hildt@pei.de](mailto:eberhard.hildt@pei.de); +49 6103 77 2140

**General changes and comments**

We thank the editor and the reviewers for their constructive comments that encouraged us to perform a careful and thorough revision of our manuscript.

Anja Schollmeier, Robin Oliver Murra, Gert Carra and Liliana Fernandes da Costa were added as co-authors

- The affiliation of Xingjian Wen was changed to “Paul-Ehrlich-Institut, Department of Virology, Langen, Germany”
- We changed and improved the abstract of the manuscript
- The manuscript was improved by thorough proofreading of a native speaker

**Reviewer 1**

Comments and answers

1.1. *Generally there is a lack of controls - for example in Fig 1 is there a control to show that GBP1 is specifically induced by HCV infection?*

We agree with the reviewer and included additional controls which indicate that GBP1 is specifically induced by HCV infection. Infection experiments in Huh7 and Huh7.5 cells analyzed by qPCR, Western Blot and imunofluorescence analyses clearly demonstrate the upregulation of GBP1 in HCV positive cells. In addition, we included Western blot analyses of stable HCV replicating cells and the GND control cells. These data have been included in Fig 1 and the corresponding figure legend of the revised manuscript. Respective changes of the main text in the results section have been added in line 283-292 and line 891-916 of the revised manuscript. This was further commented in the discussion section in line 530-534 of the revised manuscript.

1.2. *It is hard to equate the different effects of HCV on GBP1 expression - in Fig 1 HCV increases GBP1 but in Fig 2 it reduces it. The converse is also true - the effect of GBP1 on HCV is discrepant. eg GBP1 silencing has no effect on Core or RNA but decreases NS3 expression (Fig 3E).*

We apologize for the confusing description of Fig 2B and C. Fig 2 shows that the IFNγ-dependent induction of GBP1 is impaired in HCV positive cells as compared to the HCV negative cells. For a better understanding, we modified the main text in the results of the revised manuscript in line 303-305 as requested by the reviewer.

With regard to Fig 3E, GBP1 silencing decreases NS3 levels without having an effect on the NS3 distribution. However, for Core we observe a strong accumulation in the perinuclear region as depicted in the immunofluorescence staining. This is stated in the revised manuscript in line 344-352.

1.3. *I would like the see the absolute values for virus titre and RNA levels in Fig 4. Fold changes may hide interesting data.*

As requested by the reviewer we changed the values for the viral titers to absolute values. In addition, all statistical analyses in Fig 4 were re-analyzed by one-way ANOVA as recommended by reviewer 3. These changes have been included in Fig 4 and the corresponding figure legend of the revised manuscript (line 1001-1003).

**Reviewer 2**

Comments and answers

*1*	***It has been previously reported that GBP1 binds to actin and influence actin filament reorganization (e.g., PMID 24190970). As actin has a role in the shaping of autophagosomes (PMID 27723370), this must be controlled by co-staining of actin in some of the experiments of Fig.3,4,5 and discussed.***

As requested by the reviewer we performed a costainng of actin and GBP-1 to study the impact of GBP1 on actin reorganization should in our system. Therefore, we performed co-stainings of actin after IFNγ-treatment in the HCV-positive and HCV-negative cells. Indeed, we found GBP1 and HCV core colocalizing with actin filaments in the untreated HCV-positive cells (Jc1) However, we could not observe any impact of IFNγ‑treatment on the actin structures in the Jc1 or GND cells. In addition, we analyzed the impact of GBP1 silencing on the actin cytoskeleton after IFNγ-treatment. Again, we could not detect any impact of GBP1 modulation ond the actin filaments. These data have been included in Fig 2 and and Fig 3 and the corresponding figure legends(line 937-948) and (line 976-979) of the revised manuscript. Respective changes of the main text in the results section have been added in line 315-320 and line 353ff of the revised manuscript. This was further considered in the discussion section in line 590-603 of the revised manuscript.

2 *Key experiments in Fig. 1 and Fig. 3F must be validated using infectious HCV in cell culture. This would allow comparing the effect in uninfected and infected cells side by side.*

As requested by the reviewer, in the revised version of the manuscript we included infection experiments in Huh7 and Huh7.5 cells that allow the comparison of GBP1 levels in uninfected and HCV‑infected cells side by side. qPCR, Western Blot and CLSM analyses of the respective experiments clearly demonstrate the upregulation of GBP1 in HCV positive cells. These additional data have been included in Fig 1 and the corresponding figure legend line of the revised manuscript (line 893-905). Respective changes of the main text in the results section have been added in line 284-287 of the revised manuscript.

3. *Fig. 1B, 2D: an additional virus known to induce GBP1 should be shown as positive control. Ideally a virus,that infects the same cell line, e.g., VSV or HEV.*

As suggested by the reviewer we analyzed an additional virus known to induce GBP1. Based on previous results of our group, we included qPCR and CLSM-analyses of ZIKV-infected A549 cells showing a significant upregulation of GBP1 on RNA and protein level. These new data have been included in Fig 1 and the corresponding figure legends (line 909-916) of the revised manuscript. Respective changes of the main text in the results section have been added in line 291ff of the revised manuscript. This was further commented in the discussion section in line 529-534 of the revised manuscript.

4. *Fig.1D: Was only one patient being analysed? The number of patients, slides and cells analyses must be clearly described in the figure legend.*

As requested by the reviewer, we clarified this point in the revised version. This is described on line 905-909 of the revised ms The figure shows a representative staining for liver samples derived from patients suffering from chronic HCV infection.

5. *Fig. 3F: The number of data points in the CTCF graph and the statement in the legend >3 biological replicates + the number of cells per replicate analyses do not match and require clarification.*

We apologize for the unclear statement. This has been modified in the revised version of the manuscript (line 961-979).

6. *Fig. 4C: Statistics must be provided for the quantification of lysotracker/LAMP2 including error bars.*

As requested by the reviewer we provide statistics for the quantification of the LysoTracker/LAMP2 ratio. In addition, all statistical analyses in Fig 4 were re-analyzed by one-way ANOVA as recommended by reviewer 3. These changes have been included in Fig. 4 and the corresponding figure legend (line 1017-1019) of the revised manuscript.

7. *What is the effect of inhibitors / activators of autophagy on the GBP1 LAMP2 axis? Can an autophagy inducer like inhibitors of Akt or mTOR rescue the effect observed in Fig. 4C?*

As suggested by the reviewer we have included further experiments. To investigate, whether the observed effect of GBP1 silencing on the lysosomal acidification can be rescued by induction of autophagy we treated the cells with Rapamycin. Rapamycin treatment was able to increase the LysoTracker‑LAMP2 ratio after GBP1 silencing. These data have been included in Fig 4 the corresponding figure legends of the revised manuscript (line 1009-1018). Respective changes of the main text in the results section have been added in line 388-395 of the revised manuscript. This was further commented in the discussion section in line 624-628 of the revised manuscript.

8. *A positive control validating leupeptin function in this experimental setting must be shown.*

As requested by the reviewer we included data demonstrating the functionality of leupeptin under the chosen experimental conditions. For this purpose CLSM analysis with p62 as control was performed. These data are shown as Supplementary Fig 5*.*

9. *Fig. 6A: The NS3 level in the representative Western blot is not reflected by the respective quantification in the bar graph. Is the NS3 “increase” in the Western blot rather due to changes of actin expression (see major comment 1)? An alternative control instead actin is required.*

For clarity reasons we now included Western blots that clearly show an increased NS3 signal after GBP1 overexpresssion. As an alternative loading control we now used GAPDH as suggested by the reviewer. These data have been included in Fig 6 and the corresponding figure legends (line 1042) of the revised manuscript.

10. *The authors demonstrate a co-localization of core and GBP1 around the lipid droplets of in the membranous web. It has been previously demonstrated that HCV restricts access to the membranous web through nuclear pore-like structures and nuclear import machinery (PMID 26863439). Does GBP1 possess a nuclear localization signal? It seems to be able to shuttle into the nucleus based on prediction tool. This should be discussed.*

Based on prediction tools GBP1 does not harbor a classical NLS. However, HCV core contains putative NLS sequences. Based on the described colocalizaiton of GBP1 and Core we assume that Core facilitates the access of GBP1 to the MW. In addition, GBP1 has been described to directly bind to PI4P. In the HCV infected cells, PI4P accumulates in the MW to ensure high cholesterol levels. Thus, PI4P might additionally mediate the access of GBP1 to the MW. We commented on this in the revised discussion section in line 575-589 of the manuscript.

**Reviewer 3**

Comments and answers

1. *The HCV-JFHI infection cell culture experiments are carried out in Huh7.5, which are RIG-I mutated and lack type-I-IFN response. It is important to use HepG2 cells or Huh7 cells (use HCV-H77S virus) for better clarity.*

As suggested by the reviewer we included infection experiments in Huh7 and Huh7.5 cells analyzed by qPCR, Western Blot and CLSM analyses. The experiments clearly demonstrate the upregulation of GBP1 in HCV positive cells. These data have been included in Fig 1 and the corresponding figure legend (line 895-905) of the revised manuscript. Respective changes of the main text in the results section have been added in line 285-288 of the revised manuscript.

2. *All experiments in Fig 1 are done in JC1 stable expressing cell line. It is important to investigate the protein levels of GBP1 by western blotting upon HCV infection in an uninfected cell line.*

We agree with the reviewer and included Western blot analyses of Huh7 cells infected with HCV. Analyses of the corresponding blots reveal a 1.5-fold increase of GBP1 protein amounts. It should be considered that a limited number of cells are infected and that the observed effect is “diluted” by uninfected cells. These data have been included in Fig 1 and the corresponding figure legend (line 895-905) of the revised manuscript. Respective changes of the main text in the results section have been added in line 285-288 of the revised manuscript.

3. *Also, the time kinetics of GBP1 expression during HCV infection in above cell types (with JFHI, JCI and H77S viruses) will be informative for better correlation.*

As requested by the reviewer we performed infection kinetics in Huh7 cells to determine time kinetics of GBP1 expression. CLSM analyses and calculation of the CTCF indicates an increases GBP1 signal after HCV infection. This was strongest after 48 h. These images have been included in the revised Fig. 1 and the corresponding figure legends (line 901-905). Respective changes of the main text in the results section have been added in line 286-288 of the revised manuscript.

4. *In Fig 1D, the liver sections from HCV infected patients show an increase in GBP1 expression. Authors need to mention the HCV genotype of patients used in the experiments and co-relate with their experiments.*

As requested by the reviewer, we clarified this point in the revised version. The genotype was genotype 1. This is described on line 905-909 of the revised ms The figure shows a representative staining for liver samples derived from patients suffering from chronic HCV infection.

5. *In Fig 2A, the independent biological replicates should be at least n=3 (for IFN-α & IFN-β only n=2). It is necessary to analyze the experiments at same biological replicates.*

We agree with the reviewer and repeated the experiments for the IFNα and IFNβ treated cells (N=5). These data have been included in Fig 2 and the corresponding figure legend (line 922-924) of the revised manuscript.

The corresponding data for HuH7 cells are shown in the revised supplement Fig. 2.

6. *For Fig 2 B to 2D, the relative difference between Untreated cells with GND or JC1 also needs to be depicted in the graph to analyse the effect of only JC1. Possibly the basal levels of GBP1 are higher in JC1 cell line and therefore the further increase upon IFN induction is lesser. These experiments can also be repeated with virus infection in place of the use of stable cell lines.*

We thank the reviewer for pointing out that Fig 2 seems to be misleading and we clarified this point. Fig 2 aims to constitute that the IFNγ-dependent induction of GBP1 is impaired in HCV positive cells as compared to the HCV negative cells. Therefore, the graphs show the mean fold change relative to the untreated cells (GND and Jc1), set to 1. The data regarding the relative difference between untreated cells for Jc1 and GND are shown in Fig 1A and B.

7. *Fig 2A-C, upon IFN-γ treatment under GND conditions, there is more GBP1 promoter activity, mRNA level (even though they are not significant). It seems that IFN-γ + GND together increases GBP1 expression. Need to clarify.*

Indeed, as shown in Fig 2B and C, IFNγ-treatment strongly induces GBP1 expresssion in Jc1 and GND cells. However, the IFNγ-dependent induction of GBP1 is impaired in HCV-positive cells. This observation might be due to fact that HCV has evolved strategies to counteract IFN‑dependent signaling processes.

8. *Fig 2D and 3A (scr RNA-GND), the expression of GBP1 is nearer (prominent) to the peri-nuclear space while this effect is less (diffused) during HCV-JC1 infection condition. Also, not all cells show increased expression of GBP1 during JC1 expression (core). This can be clearly seen in the merged section as well. Results are confusing!*

We agree with the reviewer, low‑magnification images (40x objective) do not allow the interpretation of a subcellular localization. Due to that, we included additional high‑magnification images (93x objective) of IFNγ-treated Jc1 and GND cells to more precisely define the changed GBP1 localization to the perinuclear space in the Jc1 cells. These images have been included in Fig. 2. The corresponding figure legends (line 938-948) of the revised manuscript were adapted. Respective changes of the main text in the results section have been added in line 311-314 of the revised manuscript.

Indeed, in Fig 2D not all cells show an increased GBP1 expression in the Jc1 cells. However, these cells are treated with IFNγ and do not reflect the basal conditions of an HCV infection.

9. *In Fig 3A, under GND UT conditions, the GBP1 expression is defined around the peri-nuclear space while under Jc1 UT conditions, the expression of GBP1 is more diffused- the change in the expression of GBP1 is expected? It would be better to show GBP1 expression under GND-UT conditions compared with JC-1 UT conditions.*

We agree with the reviewer, low‑magnification images (40x objective) do not allow the interpretation of a subcellular localization. To clearly demonstrate the changed GBP1 localization in Jc1 cells, we included additional high‑magnification images (93x objective) of IFNγ-treated Jc1 and GND cells. These images have been included in Fig 2 and the corresponding figure legends of the revised manuscript (line 938-947). Respective changes of the main text in the results section have been added in line 310-320 of the revised manuscript.

10. *In Fig 3B, the densitometry does not match with the representative western blots. The expression of GBP1 under siGBP1-IFN-γ is shown less in densitometry than siGBP1-UT conditions. Re-analysis of the western blot data is required.*

We thank the reviewer for pointing out that the densitometry reflecting the GBP1 signal seems to be misleading. In Fig 3B the GBP1 graph shows the mean fold change relative to the untreated cells. Here, the untreated GND and Jc1 are set to 1. Thus, it seems as if the densitometry does not fit to the Western blots.

11. *In Fig 3D, quantification of only 4 cells might provide an erratic view. This number needs to be increased to justify the observation.*

As requested by the reviewer we increased the number of analyzed cells to 9. Respective changes are added to the figure legends (line 967) of the revised manuscript.

12. *In Fig 3E, instead of using unpaired t-test, it would be better to use one-way ANOVA analysis between the scRNA and siGBP1 (UT and IFN-γ). Is there any role of GBP1 upon HCV replication, since the authors have shown the expression of NS3 decreasing under si-GBP1 (IFN-γ) condition?*

As recommended by the reviewer we re-analyzed Fig 3 by one-way ANOVA. Regarding the impact of GBP1 silencing seems to have no impact on HCV replication as we see no impact on intracellular viral genomes after GBP1 silencing shown in Fig 3G and in line 980-982 of the revised manuscript.

13. *Fig 4B, authors have shown that upon siGBP1, core co-localize with LAMP2, what happens to NS3 in the same condition? Again, in Fig4C-D, the analysis used is un-paired t-test, instead one-way ANOVA should be used.*

As suggested by the reviewer we re-analyzed Fig 4 by one-way ANOVA. To illustrate the impact of GBP1 silencing on NS3 we included additional 3D reconstructions. In line with the reduced NS3 levels upon GBP1 silencing we found NS3 within lysosomal structures. This image has been included in Fig 4 and the corresponding figure legends of the revised manuscript (line 1008-1019). Respective changes of the main text in the results section have been added in line 368-370 of the revised manuscript. This was further commented in the discussion section in line 600ff and 624ff of the revised manuscript.

14. *Fig 4F, could this decrease in specific infectivity be linked to the decrease in Core protein levels and inefficient packaging?*

As discussed by the reviewer this could be the case, as specific infectivity is defined as the ratio of infectious viral particles (TCID50/ml) to viral genomes (genomes/ml). Lack of core affects this ratio. This is described on line 386ff of the revised ms.

15. *Fig 5B, the LAMP structures mentioned previously in Fig 4B to be induced upon siGBP1 are now visible upon IFN treatment which increase GBP1. Need to elaborate on this.*

Indeed, we observe an increased LAMP2 signal in the HCV infected cells after IFNγ treatment. This is in contrast to the literature that reports of an impaired autophagy and LAMP1/2 signal after IFNγ-treatment in A549 cells. LAMP2 is an essential factor involved in maturation of autophagosomes. HCV hijacks the autophagosomal pathway for efficient release of viral particles and is involved in several steps of the HCV life cycle. However, further experiments will be required to determine the link between IFNγ-dependent GBP1 expression and LAMP2 maturation that is essential for the HCV morphogenesis. This is addressed on line 620ff of the revised manuscript-

16. *In Fig 6, the authors have shown that upon GBP1 over-expression, HCV-core protein co-localizes with GBP1 at peri-nuclear space, what happens during siRNA conditions?*

These data are shown in figure 3 and described on line 325ff of the revised manuscript. Silencing of GBP1 leads to intracellular accumulation of core and to a reduction of NS3.

17. *Fig 6. What happens to extracellular RNA levels and intracellular RNA titres upon GBP1 overexpression? This is essential to be compared with fig 4 D-F data to define an inverse correlation upon silencing or overexpression of GBP1 and to specify the role of GBP1 on packaging or release of the virus particles.*

As requested by the reviewer we extended these analyses and included additional data describing ther impact of GBP1 overexpression on the amount of extracellular RNA. We observe a significant increase in the number of released infectious viral particles and released viral genomes. Indeed, this reflects that overexpression of GBP1 favours release of viral particles which is associated with a decrease of intracellular viral genomes. This is described on line 435ff of the revised manuscript and shown as additional part of figure 6 (fig. c, line 1048-1052).

18. *Fig 6E. and 3C. Both silencing and overexpression of GBP1 leads to perinuclear core aggregation. Not clear why?*

The GBP1 overexpression favours viral release and thereby leads to increased amount of extracellular viral RNA. This is associated with a decreased amount of core as evidenced in figure 6a. Core colocalizes in vesicular structures with GBP1 upon overexpression of GBP1. This is shown in figure 6F of the revised manuscript (line 1054-1058). In contrast to this an intracellular accumulation due to an impaired release of core occurs leading to lagrge unsoluble core aggregates as shown in figure 3c of the revised manuscript. A reason might be the impaired assembly that prevents virus morphogenesis and release.

19. *Fig 7F, the observation is very interesting. However, it is intriguing why such structures of GBP1 were not visible in intracellular GBP1 staining in the previous experiments and also in the experiment 7G? It would be important to quantify the percentage of cells exhibiting such staining in this context.*

There are two aspects that have to be considered: The one aspect is that in contrast to the previous experiments here GBP1 is overexpressed. The endogenous GBP1 levels are so low that it is barely detectable. The second point is that only 20% of LDs are coated with core as (Myanari et al., 2007, Nature cell biology). This is described on line 547 ff of the revised manuscript.

20. *In Fig 7G, authors have used Huh9-13 replicon model for showing the co-localization of GBP1, dsRNA and LDs. A better experiment would be, over-expression of core protein in Huh9-13 cells and then see if the association still exists.*

As suggested by the reviewer we included experiments with pcCore transfected Huh9-13 cells. Indeed, we see a clear colocalization of Core and GBP1 in the transfected cells. In addition, pcCore transfection resulted in the same GBP1 phenotype as in the Jc1 cells were GBP1 completely coats the LDs which further corroborates our hypothesis that core is essential to target GBP1 to LDs. This image has been included in Fig 7 (Fig 7I)and the corresponding figure legends of the revised manuscript (line 1086-1091) . Respective changes of the main text in the results section have been added in line 484-486 of the revised manuscript. This was further commented in the discussion section in line 545 ff of the revised manuscript. In addition, Fig 7G-I have been rearranged.

21. *Fig 8, the graphical abstract can be made clearer by proper labelling the pathway as a, b, c, etc*

We agree with the reviewer and labelled the different pathways in the graphical abstract with number 1-4.

Minor

1. Reviewer #2: 1. Fig. 6E: the magnification of the additional zoom window within the inset should be stated in the legends.

This information has been included in the figure legend (6F of the revised ms)

2. Material and Methods: the use of upper and lower cases in the subtitles.

This has been corrected.

3. Typo line 230: “4°C.bHorse..”

The typo has been eliminated.

4. The order appearance of Figure panels should be respected: Fig. 4C cited before Fig. 4B

2. Reviewer #3:

 1. As a comparison of Fig 1 B and 1 C, the localisation of GBP1 seems to be inconclusive. In 1C, it seems cytoplasmic but in 1B, the staining is very faint and not cytoplasmic. Please comment on this or provide a better representative image.

This might be a misunderstanding, figure 1B is a promoter analysis and 1C an immunofluorescence analysis.
2. The manuscript requires extensive English and grammatical corrections.

The ms was read and corrected by a native speaker
3. Difference in referencing style in discussion section, line no: 473-474

This was corrected
4. Grammatically wrong sentence, line 519-521.

This was corrected
5. In the material and method section, several sentences are abruptly ending, (for e.g., line 89-90). Also, figure legend 5, sentence is abruptly ending.

We corrected this
